# Nanomaterial-Based Antivascular Therapy in the Multimodal Treatment of Cancer

**DOI:** 10.3390/pharmaceutics15041207

**Published:** 2023-04-11

**Authors:** Xiaocong Ma, Weimin Fang, Duo Wang, Ni Shao, Jifeng Chen, Tianqi Nie, Cuiqing Huang, Yanyu Huang, Liangping Luo, Zeyu Xiao

**Affiliations:** 1 The Guangzhou Key Laboratory of Molecular and Functional Imaging for Clinical Translation, The First Affiliated Hospital of Jinan University, Guangzhou 510632, China; 2 The 12th People’s Hospital of Guangzhou, Guangzhou 510620, China; 3 Department of Ultrasound, Guangdong Women and Children Hospital, Guangzhou 511400, China; 4 Department of Biochemistry and Molecular Medicine, University of California Davis, Sacramento, CA 95817, USA

**Keywords:** antivascular therapy, nanomaterials, chemotherapy, immunotherapy, phototherapy

## Abstract

Abnormal tumor vasculature and a hypoxic tumor microenvironment (TME) limit the effectiveness of conventional cancer treatment. Recent studies have shown that antivascular strategies that focus on antagonizing the hypoxic TME and promoting vessel normalization effectively synergize to increase the antitumor efficacy of conventional therapeutic regimens. By integrating multiple therapeutic agents, well-designed nanomaterials exhibit great advantages in achieving higher drug delivery efficiency and can be used as multimodal therapy with reduced systemic toxicity. In this review, strategies for the nanomaterial-based administration of antivascular therapy combined with other common tumor treatments, including immunotherapy, chemotherapy, phototherapy, radiotherapy, and interventional therapy, are summarized. In particular, the administration of intravascular therapy and other therapies with the use of versatile nanodrugs is also described. This review provides a reference for the development of multifunctional nanotheranostic platforms for effective antivascular therapy in combined anticancer treatments.

## 1. Introduction

Tumor neovascularization is essential for the growth of solid tumors [1]. Tumor growth and metastasis require the constant creation of new blood vessels, which enables cells to obtain nutrients and oxygen [2]. The following can lead to the formation of a hypoxic and acidic microenvironment with high interstitial fluid pressure (IFP) in tumors: the leakage, curvature, and dilation of blood vessels in neoplasms; morphological abnormalities of endothelial cells (ECs); loose pericellular junctions; and the absence of pericellular cells [3,4,5].

In recent years, according to the abnormal characteristics of tumor blood vessels, three types of antivascular strategies have been proposed: (1) The anti-angiogenic strategy uses angiogenesis inhibitors to inhibit the growth of vascular smooth muscle cells, block the formation of vascular networks, and inhibit EC proliferation and migration from achieving anti-angiogenic effects, thereby effectively controlling tumor growth [4,6,7]. (2) The vascular destruction strategy aims to destroy the ECs of existing tumor blood vessels through vascular disrupting agents (VDAs), resulting in the obstruction of blood flow, thus blocking tumor cell metastasis [8]. The most commonly used VDA in clinical practice is combretastatin A4 (CA4). CA4 is a tubulin-binding agent that destroys the vasculature by selectively targeting and destroying established tumor vascular ECs, causing tumor vascular blockage and leading to the ischemic necrosis of tumor tissue [5,9,10]. (3) Vascular blockade therapy aims to restrict blood flow in vessels by inducing the formation of blood clots or gel phase transitions within blood vessels, thereby blocking the supply of blood, nutrients, and oxygen to the tumor [11]. Three different blocking pathways can be activated: the thrombin activation pathway, the fibrin activation pathway, and the platelet activation pathway [12]. However, single-agent antivascular therapy does not have a prominent antitumor effect. Cancer cells can develop resistance to anti-angiogenic drugs via different mechanisms, such as the acquisition of pro-migration phenotypes or the upregulation of the levels of other angiogenic molecules. For example, treatment with bevacizumab, which targets vascular endothelial growth factor (VEGF), may lead to a promigratory phenotype in drug-resistant glioblastoma [13,14,15]. Vascular destruction therapy can induce hypoxia and thus lead to tumor recurrence; it is only effective in inducing the destruction of the central tumor tissue and ignores the peripheral blood vessels of the tumor [9]. Vascular blockade strategies are often limited by the recurrence of residual tumor cells. For this reason, strategies that combine vascular-targeted therapy with existing therapies may achieve synergistic effects. Much of the recent research on vascular therapy has focused on novel biomaterials, such as nanomaterials. Nano-assisted tumor vascular therapy may improve the overall quality of vascular care, and the use of nano-targeted drug delivery may offer improved treatment strategies for tumor vasculature.

**Figure 1:**
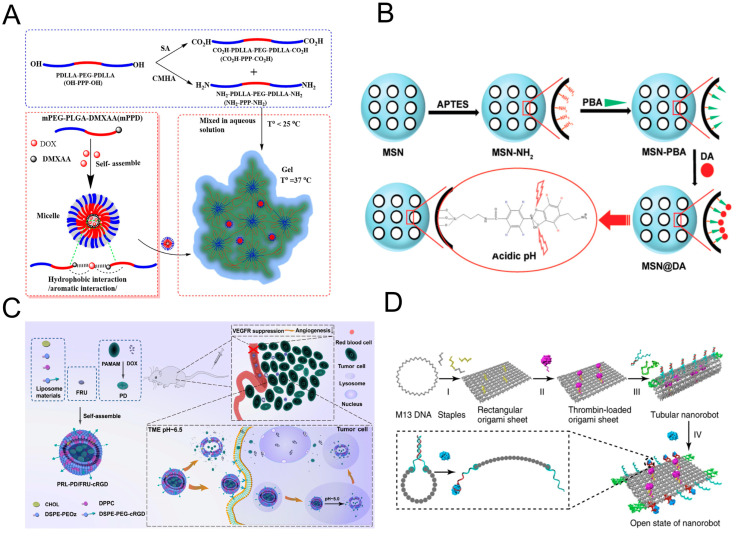
Antivascular therapy combined with nanoparticles to enhance chemotherapeutic drug delivery. (**A**) Schematic representation of hydrogel-based dual drug delivery for local treatment. Adapted with permission [26]. Copyright © 2023 Elsevier Ltd. (**B**) MSNs synthesized, functionalized, and loaded with NH2, PBA, and DA, respectively. These MSNs have a pH-responsive bond between PBA and DA that enables them to release the drug in acidic pH upon arrival in the tumor microenvironment. Adapted with permission [28]. Copyright © 2023 WILEY-VCH Ltd. (**C**) Antitumor mechanism of PRL-PD/FRU-cRGD. Adapted with permission [29]. Copyright © 2022 American Chemical Society. (**D**) Design and characterization of a thrombin-functionalized DNA nanorobot. Adapted with permission [30]. Copyright © 2018 Nature Publishing Group.

Some nanomaterials are specially designed to aggregate at tumor sites to improve cancer diagnosis and treatment, as well as to detect tumor response to treatment and improve prognosis [16,17]. Many new nanocomposites have been developed as drug delivery carriers capable of improving drug delivery efficiency and reducing systemic toxicity [18,19]. Regarding nanoparticles, it has been shown that the conjugation of targeted ligands can achieve precise drug delivery and overcome biological barriers to enable the drug to reach the tumor core [20,21]. In addition, some nanomaterials themselves can also achieve the purpose of targeting tumor blood vessels, thus resolving the problems encountered in vascular therapy [22,23,24,25]. Given the ease with which triblock copolymers can be formed, Darge et al. designed a triblock copolymer hydrogel (PDLLA-PEG-PDLLA) based on the sol–gel transformation, and this hydrogel can achieve a continuous high concentration of drug at the tumor site, reducing the toxicity associated with chemotherapeutic drugs, such as DOX, and the systemic administration of VDAs to human organs (Figure 1A) [26]. Some nanomaterials are responsive to pH, redox potential, GSH level, and light and thus can be used for the controlled release of cargo after exposure to different stimuli [27]. Taleb harnessed the high loading capacity and excellent stability of mesoporous silicon nanoparticles (MSNs) as a carrier for DA (a chemical messenger that inhibits angiogenesis) delivery, as MSNs can be easily modified to have pH-sensitive bonds for targeted delivery to the acidic microenvironment of tumors (Figure 1B) [28]. The nanodrug delivery system designed by Zhang’s team (PRL-PD/FRU-cRGD) can be modified to specific sizes (large, ~120 nm; small, ~15 nm) and easily reach the core of the tumor to exert strengthened antitumor effects (Figure 1C) [29]. Nie Guangjun’s team prepared a nanorobot that can be programmed to specifically deliver thrombin to the corresponding part of the tumor, causing tumor blood vessel embolism (Figure 1D) [30]. In addition, nanoparticles can also bind to platelet membranes as a delivery platform for antitumor drugs and therapeutics. Nie used platelet membrane-coated MSNs combined with VDAs, and the nanoparticles damaged blood vessels and tended to aggregate at the damaged vascular site, resulting in amplified vascular damage [31]. The interactions of antivascular therapy, nanotherapy, and conventional therapies have led to the birth of multimodal synergistic therapies. Alamzadeh proposed a co-loaded gold nanoparticle–cisplatin hydrogel complex for a combination of thermochemotherapy and radiotherapy [32]. Xu designed a multimodal combination therapy based on ultrasound-responsive nanoparticles [33]. Mirrahimi designed a nanotherapy platform that can be used for CT/MR dual imaging and realized DOX release and strong thermochemotherapeutic effects [34]. You developed a nanoplatform modified with Pt (platinum), which acts as a catalyst to continuously break down H_2_O_2_ to O_2_ and relieve hypoxic photodynamic therapy [35]. Lu designed a mesoporous Fe_3_O_4_ nanocomposite that improves interventional embolism when used in combination with thermal ablation and multimodal imaging [36]. 

Existing oncology treatments, such as chemotherapy, immunotherapy, and phototherapy, are limited. The increase in the IFP limits the delivery of antitumor drugs and their penetration into tumor tissue, and hypoxia reduces the sensitivity of tumor cells to drugs. In addition, the acidic microenvironment of tumors can also damage the cytotoxic function of immune cells [3]. Given the limitations of monotherapy, recent studies have focused on combination therapy to enhance anticancer effects. In this review, to provide a reference for the rational design of versatile nanomedicine-based therapies involving antivascular therapy, we systematically summarize the results of the application of different nanoplatforms in combination antivascular therapy regimens and various cancer treatments in recent years.

## 2. Immunotherapy

In recent years, immunotherapy has played a key role in tumor treatment, but its development is still hindered by many factors. Studies have shown that antivascular therapy can improve the immunosuppressive microenvironment and increase infiltration by immune effector cells. Table 1 describes existing combination antivascular therapy and immunotherapy strategies and the mechanisms of action of antivascular drugs; these strategies can achieve selective drug delivery and drug accumulation in the tumor microenvironment when combined with nanotherapy.

CD8^+^ T cells play a crucial role in antitumor immunological therapy because of their direct antitumor cytotoxic function. Programmed death 1 receptor (PD-1) is an immunosuppressive receptor located on T cells that can bind to programmed cell death-Ligand 1 (PD-L1) located on stromal cells, inhibiting the activation of T cells and making them incapable of attacking. PD-L1 is highly expressed in tumor-infiltrating lymphocytes and inhibits the immune-killing function of CD8^+^ T cells. Immune checkpoint inhibitors (ICIs) are effective as tumor treatment because they block the binding of PD-L1/PD-1. ICIs directly block the effect of PD-1 on CD8^+^ T cells, decreasing their proliferation [46,47,48]. However, there is a risk of toxic side effects with ICIs; for instance, it is easy to cause immune-mediated side effects, causing endocrine system diseases [49].

**Figure 2:**
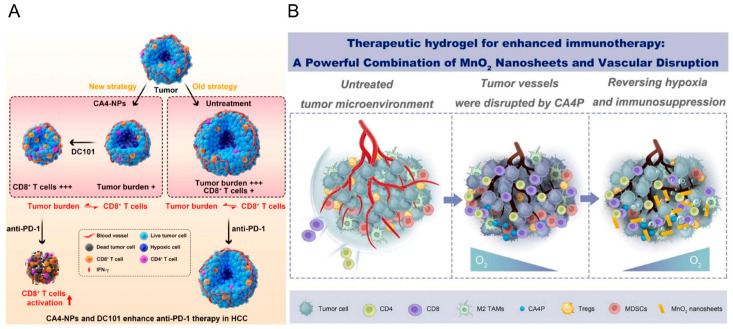
Antivascular therapy combined with nanoparticles can relieve immunosuppressive microenvironment. (**A**) MSNs synthesized, functionalized, and loaded with NH2, PBA, and DA, respectively. MSNs have a pH-responsive bond between PBA and DA that enables them to release in acidic pH upon arriving in the tumor microenvironment. Adapted with permission [41]. Copyright © The authors. (**B**) The therapeutic hydrogel (CM@Gel) combines with a vascular disrupting agent, CA4P, to alleviate tumor hypoxia after selective blockade of tumor nutrient sources [39]. Copyright © 2023 Elsevier Ltd. All rights reserved.

VEGF overexpression in tumors inhibits the migration of cytotoxic T lymphocytes (CTLs) and antigen presentation, thereby hindering T-cell activation and promoting the recruitment and activation of immunosuppressive cells [50]. In 2020, the FDA approved ICIs in combination with anti-angiogenic drugs for the treatment of patients with inoperable HCC or patients with HCC ineligible for transplantation [51]. Some studies have shown that antivascular strategies combined with immune checkpoint blockade can effectively improve the efficacy of immunotherapy [45,52]. VEGF can prevent T cells from infiltrating tumors by promoting endothelial nonreactivity; thus, it inhibits the anticancer effect of ICIs and leads to the apoptosis of CD8^+^ T cells. Bevacizumab significantly reduces VEGF expression in tumors by inhibiting the binding of VEGF-A to VEGF receptor-2 (VEGFR-2) [53], increasing infiltration by and the cytotoxic function of CD8^+^ T cells in tumors [54,55]. In addition, bevacizumab, in combination with ICI therapy, decreased the expression of PD-L1, showing a long-lasting antitumor effect [55]. Feng’s team designed a tumor-targeting gene complex nanoparticle that co-delivers pshVEGF-A and pshPD-L1. By downregulating VEGF-A and PD-L1 to block immune checkpoints, pshVEGF-A, as an anti-angiogenic drug, improves the tumor immune microenvironment [44]. The selective blockade of VEGFR-2 using apatinib inhibits the VEGFA-mediated proliferation and migration of ECs and enhances the antitumor effects of anti-PD-1 mAbs [56,57]. Cho established an RGD-modified lipid nanoparticle to deliver small interfering RNA (siRNA) to tumor endothelial cells (TECs) to knock down the expression of VEGFR-2. Combined with aPD-1 mAb reduces the number of tumor-infiltrating lymphocytes (TILs) and enhances infiltration by CD8^+^ T cells. This combined strategy normalizes the tumor vasculature, which successfully suppresses tumor growth [38]. Response rates in advanced HCC are limited due to a deficient number of CD8^+^ T cells due to the tumor burden. Bao designed CA4-NPs that bind to the VEGF/VEGFR-2 inhibitor DC101. CA4-NPs reduce tumor burden by selectively destroying established blood vessels. DC101 can decrease the high expression of VEGF after VDA therapy; temporarily normalize tumor blood vessels; increase the number of CD8^+^ T cells within the tumor; and significantly increase the levels of IFN-γ, TNF-α, and IL-2 after treatment. Having a number of CD8^+^ T cells that is proportional to the tumor burden enhances the efficacy of anti-PD-1 therapy (Figure 2A) [41]. 

Increasing evidence shows that the tumor immune microenvironment (TIME) largely determines the treatment outcome of cancer immunotherapy and plays a nonnegligible role in tumor immune monitoring and immune avoidance [58,59,60]. Antivascular therapy combined with nanostrategies can facilitate immunotherapy by improving the abnormal microenvironment of tumors [61,62,63,64,65]. Zhou designed a nanoplatform using axitinib (a tyrosine kinase inhibitor) that inhibited cell signaling to inhibit tumor cell growth and proliferation, promote tumor vascular normalization, and overcome immunosuppression, enhancing the transport of immune cells into the tumor parenchyma and improving the effect of immunotherapy [37]. Huang established AuNPP-FA, AuNPs that can inhibit endothelial Smad2/3 signaling, increase pericyte coverage, and upregulate VE-cadherin on ECs by upregulating SEMA3A and downregulating VEGF-A expression to strengthen tight junctions and normalize tumor vasculature. Increased infiltration by CD3^+^ and CD8^+^ T cells in tumors improves immunotherapy [43]. Taleb designed a bifunctional peptide-assembled nanoparticle composed of an anti-angiogenic peptide (FSEC) and an immune checkpoint-blocking peptide (DPPA). FSEC induced significant anti-angiogenic effects in a mouse model of breast cancer, debulking blood vessels to allow adequate infiltration by immune cells to be achieved and restore immunosuppression in the TME [42]. Luo’s team designed a therapeutic hydrogel simultaneously loaded with MnO_2_ nanosheets and the vascular-destroying agent combretastatin-A4 phosphate (CA4P). On the one hand, CA4P blocks the nutrient oxygen supply of the tumor by destroying the ECs of the tumor vasculature and alleviates the immunosuppression caused by hypoxia. On the other hand, MnO_2_ nanosheets react with hydrogen peroxide (H_2_O_2_) within tumors to produce oxygen to alleviate the tumor hypoxia caused by CA4P. This highly effective combined method can also activate host immune responses by recruiting immature dendritic cells into tumors, increase intratumor infiltration by CD4^+^ and CD8^+^ T cells, and significantly enhance the efficacy of a-PD1 therapy (Figure 2B) [39]. Zhou synthesized a polymeric copper chelator that inhibits angiogenesis by inducing copper deficiency. It was designed as a nanoparticle that can achieve controlled release and targeted transport. This combined approach enhances immune activation in breast cancer [40]. Current research focuses on anti-angiogenic strategies that limit antigen presentation and VEGF-inhibited T-cell activation by inhibiting VEGF expression.

Vascular therapy combined with immunotherapy can inhibit the formation of new blood vessels or destroy existing blood vessels to block the nutrient and oxygen supply of tumors, reverse the inhibition of T cells induced by the immunosuppressive microenvironment, and increase infiltration by lymphocytes. In addition, some antivascular drugs combined with immunotherapy can decrease the expression of PD-L1, enhance sensitivity to ICIs, and exhibit increased antitumor effects.

## 3. Chemotherapy

Chemotherapy is one of the main treatments for cancer. Chemotherapeutic drugs can eliminate cancer cells and inhibit tumor growth, and cancer patient survival time can be prolonged with chemotherapy in the early stage, but as the disease progresses, chemotherapy resistance commonly develops, increasing the likelihood of recurrence and metastasis [66,67]. Studies have shown that antivascular therapy can solve the challenges of blood perfusion and high IFP in tumors, prolong the half-life of chemotherapy, and improve the efficiency of drug delivery. Table 2 describes existing antivascular therapies combined with chemotherapy strategies and the mechanisms of action of the included antivascular drugs.

**Figure 3:**
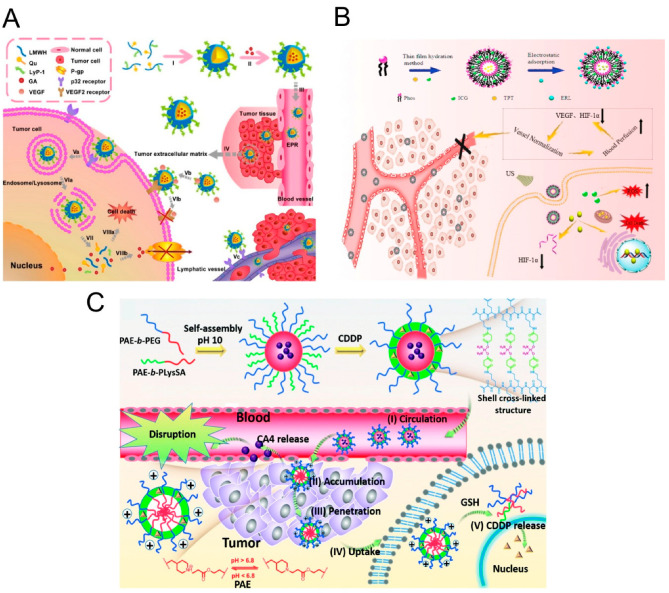
Antivascular therapy combined with nanoparticles enhances chemotherapeutic drug delivery. (**A**) NPs@DA can affect endothelial cells and pericytes to normalize the structure of vessels and enhance the delivery of chemotherapeutic drugs to tumor cells. Treatment with the pH-sensitive NPs@DA system enhances the effect of therapy in mouse tumor models. Adapted with permission [74]. Copyright © 2018 Elsevier Ltd. (**B**) Schematic illustration of efficient coencapsulation of PTX and SB into Dex-DOCA amphiphilic polymers and the use of PTX + SB NPs as a robust nanoplatform, which achieves prolonged circulation, eradication of stromal components, and normalization of tumor vessels for enhanced drug accumulation and efficacy in solid tumors. Adapted with permission [73]. Copyright © 2020 American Chemical Society. (**C**) Schematic illustration of the preparation of DRN and the shell crosslinking structure, including a description of programmable drug release characteristics in vivo. Adapted with permission [70]. Copyright © Royal Society of Chemistry.

The IFP in tumors is higher than that in normal tissues, which compresses blood vessels and inhibits the delivery of chemotherapeutic drugs. Therefore, reducing the IFP by normalizing the vasculature is key to enhancing the efficacy of chemotherapy [5]. Some current studies are using nanocarriers to achieve the combined application of antivascular drugs and chemotherapeutic drugs [72,78,79,80,81,82,83,84]. It has been reported that moderate-dose anti-angiogenic drugs can kill small nonfunctional blood vessels to normalize the tumor vasculature, resulting in increased the accumulation of and penetration by the chemotherapeutic drug and a significant improvement in the oxygen concentration within solid tumors [85]. Zhang designed a pH-triggered size-switchable nanodrug delivery system loaded with fruquintinib, which inhibits angiogenesis by binding VEGFR-1, -2, and -3 and reducing the IFP, and the system overcomes the problem of the poor permeability of large nanoparticles in tumor tissue and achieves deep delivery of DOX into breast cancer tissue [29]. Taleb constructed mesoporous silicon nanoparticles (MSNs) and utilized a pH-sensitive bond between DA and phenylboronic acid (PBA) to encapsulate DA in synthetic MSNs to make release in the tumor’s acidic microenvironment possible. Alternatively, increasing blood flow perfusion, reducing IFP, is an effective strategy that can improve the efficacy of chemotherapy by increasing the delivery of and penetration by chemotherapeutic drugs [28]. Low-molecular-weight heparin (LMWH) exerts an antivascular effect by binding to bFGF and VEGF. Tian developed an amphiphilic nanomaterial, the LyP-1-LMWH-Qu (PLQ) conjugate. LyP-1 can target tumors, and PLQ nanoparticles can inhibit the expression of P glycoprotein (P-gp) in tumor cells, reverse drug resistance, and inhibit tumor cell proliferation and angiogenesis. Combination chemotherapy and antivascular therapy reduce the tumor microvascular density, increase pericyclic-cell cover, and reduce the IFP, thereby promoting penetration by chemotherapeutic drugs into breast cancer tissue (Figure 3A) [74]. Du used nanomedicine lipid derivative conjugates to bind to LMWH to improve the delivery of chemotherapeutic drugs such as gemcitabine and paclitaxel [75].

A recurring problem with chemotherapy is that tumor cells are prone to developing resistance to chemotherapeutic drugs [86,87]. Multidrug resistance describes a situation in which cancer cells have developed resistance to more than one anticancer drug, despite the fact that these drugs have very different molecular structures and mechanisms (MDR) [88]. In order to treat tumors where multiple drugs have failed, some nanocarriers have been created [89,90,91]. The combination of chemotherapy with antivascular strategies has been successful in increasing the sensitivity of tumor cells to chemotherapy and effectively inhibits tumor metastasis and recurrence [92,93]. Huo used the dextran deoxycholic acid (Dex-DOCA) amphiphilic polymer as a delivery system to encapsulate paclitaxel (PTX) and silybin (SB), forming (PTX + SB) NPs with synergistic antitumor effects. SB can exhibit anti-angiogenic activity and increase the sensitivity of tumor cells to chemotherapeutic drugs by modulating the ERK, Akt, and STAT3 pathways. In addition, SB can increase PTX toxic effects in solid tumors, thereby significantly increasing drug availability in deep tumor cells. Dex-DOCA is able to deliver PTX and SB in a predetermined synergistic ratio, thereby prolonging the half-life of the drug in blood circulation and enhancing its accumulation inside the tumor [76]. Tumors metastasize and migrate through endothelial-dependent blood vessels (EDVs) that have formed in solid tumors and vascular mimicry (VM) areas that have formed because of the proliferation of highly aggressive tumor cells. Luo’s team designed a self-assembling nanoparticle (VE-DDP-Pro) that releases VE-DDP and employed both integrin αvβ3 and integrin α5β1 to modulate the AKT/mTOR/MMP-2/laminin and AKT/mTOR/EMT signaling pathways; furthermore, the knockdown of MMP-2 inhibited VEGF release, simultaneously having effects against both VM areas and EDVs, thus greatly improving the efficacy of cisplatin in ovarian cancer [71]. Chen’s team designed a polylactic acid–glycolic acid (PLGA) nanocarrier loaded with hypoxia-activated prodrug (HAP) and Vadimezan. HAP can improve Vadimezan’s vascular destruction potency, and this combination strategy enhances the efficacy of chemotherapy [94]. Restoring the permeability and perfusion capacity of the tumor vasculature can increase the ability of chemotherapeutic drugs to reach the deep sites of tumors.

Jain proposed a new concept, tumor vascular normalization (TVN), which can improve chemotherapy outcomes by reshaping the tumor microenvironment and enhancing drug delivery [95]. However, a serious challenge currently facing chemotherapy is the transient effect of TVN, which presents challenges related to the administration of chemotherapeutic drugs in the TVN window. As a solution, we need to come up with new ways to keep the TVN effect going for a longer period of time. GBM neovascularization is primarily mediated by VEGF signaling, but alternative mechanisms, such as anaerobic glycolysis, can be quickly activated as a bypass. Li developed an anionic liposome nanosystem containing the chemotherapeutic drug topotecan (TPT), the sensitizer indocyanine green (ICG), and the antivascular drug erlotinib (ERL). ERL is a tyrosine kinase inhibitor that has been found to be capable of normalizing the tumor vasculature by downregulating VEGF while inhibiting the epidermal growth factor receptor (EGFR). However, persistent anti-VEGF therapy leads to hypoxia-inducible factor-1α (HIF-1α) upregulation, which eventually leads to the development of tumor hypoxia and drug resistance. Combination with the chemotherapeutic drug TPT can effectively prevent the production of HIF-1a and ultimately prolong TVN. ICG-mediated sonodynamic therapy (SDT) can reduce the expression of VEGF. This combination strategy can prolong TVN so that chemotherapeutic drugs can have longer-lasting effects on tumors (Figure 3B) [73]. Nie’s team synthesized a chitosan-based polymer nanoparticle loaded with both DOX and thrombin. The combination of chemotherapy and vaso-blocking therapy can produce the synergistic effects of blocking the blood supply to tumors and inhibiting cancer cell proliferation [77].

VDAs cause the degradation of the basement membrane and ultimately induce the massive central necrosis of tumor tissue. Chemotherapeutic drugs are responsible for killing the tumor cells that proliferate around the lesion. The combination of a VDA and chemotherapeutic drugs has greater antitumor activity than either as a single agent, leading to extensive and broad tumor necrosis [96]. Traditional chemotherapeutic drugs easily trigger angiogenesis in the later stages of treatment, resulting in tumor metastasis recurrence. Nie’s team designed MSNs coated with platelet membranes that bind the VDA to the anti-angiogenic agent. Platelet membranes can target damaged sites of tumor blood vessels, leading to effective vascular destruction [31]. Liu developed a dual-carrier drug-targeting lamellar nanoparticle that simultaneously delivers CA4 and Dox, which target two different cell populations within the tumor; the system significantly enhanced the drug-induced killing of tumor cells in mouse melanoma models [68]. Jiang used the sequential delivery of CA4-NPs and matrix metalloproteinase 9 (MMP9) to enhance tumor therapy. CA4 can enhance the expression of MMP9 in tumor tissue by destroying immature tumor blood vessels and causing a hypoxic microenvironment that significantly promotes the release of DOX prodrugs. The combination of CA4-NPs with MMP9-DOX-NPs showed significant antitumor efficacy in situ in 4T1 tumor-bearing mouse models [69]. Ding developed a pH-lowering dual-reactive drug release system for the programmable release of CA4 and CDDP nanocarriers, which enables the release of CA4 at perivessel sites in tumor tissues to destroy blood vessels to be achieved; this cargo is absorbed by cancer cells inside the tumor tissue, and the reducing conditions surrounding cells trigger the release of CDDP and promote the apoptosis of cancer cells (Figure 3C) [70]. Darge used a VDA (DMXAA) and DOX to synergistically improve chemotherapy and hydrogels to sequentially release drugs locally, effectively inhibiting tumor growth [26].

Vascular therapy combined with chemotherapy can effectively inhibit blood flow perfusion and decrease the high IFP, which promotes deep infiltration by the chemotherapeutic drugs into tumor tissues, targets tumor blood vessels to induce vessel normalization, prolongs TVN, and increases the delivery efficiency of chemotherapeutic drugs. Antivascular drugs enhance chemotherapy sensitivity by inhibiting hematopoietic pathways. VDAs, in combination with chemotherapeutic drugs, can target different cell populations within the tumor, leading to widespread tumor necrosis.

## 4. Phototherapy

Phototherapy for tumors mainly includes photodynamic therapy (PDT) and photothermal therapy (PTT). Studies have shown that the combination with antivascular therapy can overcome the problem of insufficient reactive oxygen species (ROS) production with PDT therapy, and combination PTT therapy can increase the near-infrared absorption of tumors so that PTT therapy can kill tumor cells with less laser energy. Numerous studies have investigated the potential of combining antivascular therapy and phototherapy [97,98,99,100,101,102,103,104,105]. Table 3 describes existing strategies combining antivascular therapy and phototherapy and the mechanisms of action of the included antivascular therapies.

**Figure 4:**
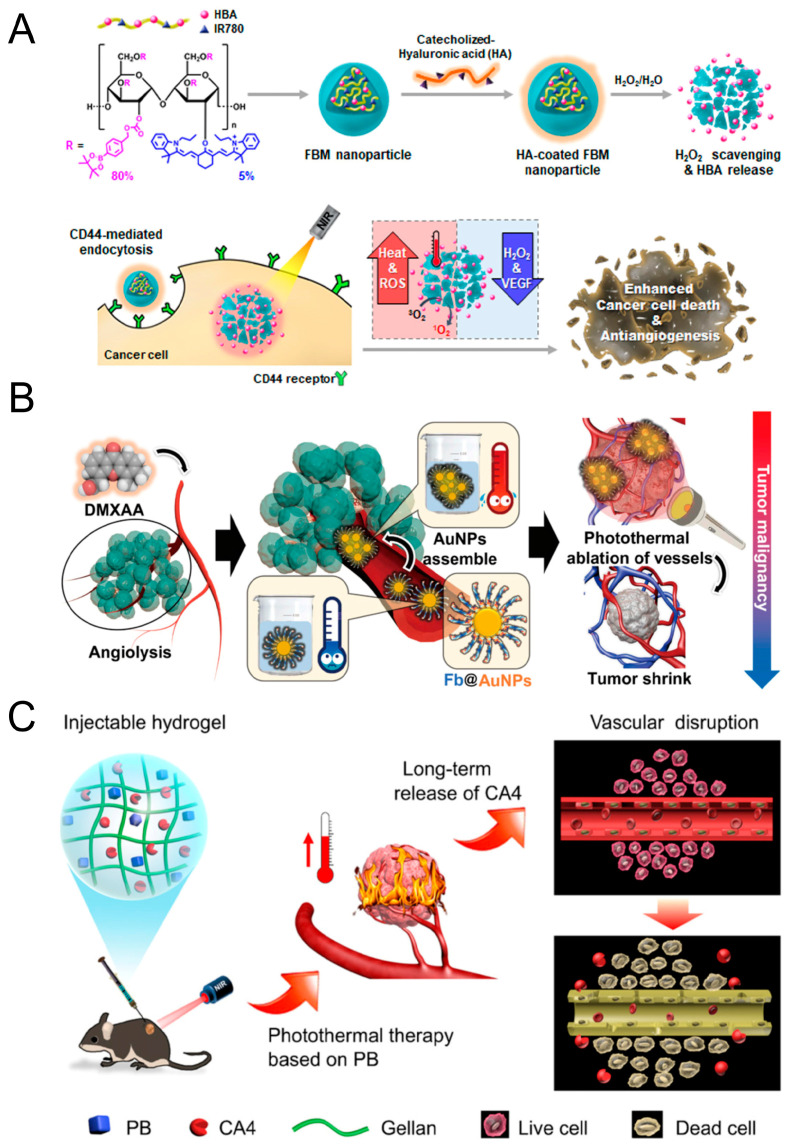
Antivascular treatment enhances the photothermal conversion efficiency of PTT. (**A**) Schematic diagram of HA-FBM nanoparticles as phototherapeutic agents. Adapted with permission [116]. Copyright © 2021 American Chemical Society. (**B**) Schematic diagram of the peTVD strategy. Adapted with permission [115]. Copyright © 2020 The authors. (**C**) Schematic illustration of an injectable NC hydrogel for the codelivery of CA4 and PB in synergistic photothermal and vascular-disrupting therapy. Adapted with permission [109]. Copyright © 2019 American Chemical Society.

PDT uses a combination of photosensitizers, light, and oxygen molecules to treat cancer and is widely used in the treatment of various diseases due to its noninvasive characteristics [119]. The highly toxic ROS produced by the energy transfer between photosensitizers and molecular oxygen lead to cell death and tumor elimination. The lethality of singlet oxygen in tumors is insufficient, resulting in a high recurrence rate after PDT treatment [120]. In addition, tumor hypoxia limits the production of ROS; thus, the therapeutic effect of PDT is limited. Tumor cells that survive PDT produce angiogenic factors and excess glutathione (GSH), deplete the ROS produced during PDT treatment, and impair the killing effect of PDT. These additional factors increase the likelihood of tumor recurrence and metastasis [119]. Numerous studies have investigated the potential of combining anti-angiogenic therapy and photodynamic therapy. When bevacizumab was administered after PDT to inhibit neovascularization and reduce the density of microvessels in the tumor, it improved the effectiveness of PDT and significantly inhibited tumor growth and recurrence [106]. Min designed multifunctional biomimetic MOF nanoparticles as carriers for PDT reagents and apatinib. In tumor tissue, a layer of MnO_2_ deposited on the MOF nanoparticles can react with glutathione to consume excess GSH. When the MnO_2_ shell is degraded, apatinib is released to neutralize PDT-induced vascular reconstruction, and this combined strategy improves the efficacy of PDT [107]. Eunkyeong Jung designed a fluorescent borate polysaccharide (HA-FBM) nanoparticle that can be heated under laser irradiation to release HBA to inhibit the expression of VEGF, thereby improving the oxygen restriction induced by PDT (Figure 4A) [116]. Type II PDT relies on singlet oxygen (1O2) produced by the photosensitizer upon irradiation. Given the hypoxia-related challenges of tumor treatment, Chen et al. focused on type I PDT based on superoxide radicals (O_2_^−^) and designed a bifunctional organic nanoconjugate (BDPVDA) as an organic superoxide radical (O_2_^−^) nanocarrier; this carrier releases the VDA to induce blood vessel rupture to cut off the oxygen supply induced by type II PDT and block tumor metastasis pathways. The contraindications of PDT and VDA treatment were thus resolved [114]. Liu designed a nanodrug (CeCA) consisting of CA4 and the photosensitizer chlorine e6 (Ce6). CA4 can enhance the vascular destruction induced by PDT, and Ce6 synergistically acts with PDT under light to produce a large amount of 1O2. CeCA increases the lethality of PDT against tumor cells and blocks EC migration and angiogenesis [108]. Liang designed acidic TME-responsive unsupported carbon nanoconjugates (DAA NPs) by combining DMXAA and a photosensitizer, which achieved complete tumor ablation [114].

PTT converts light into thermal energy to kill tumor cells and is widely used in tumors because of its noninvasive nature and high efficacy [121]. The inefficient conversion of photosensitizer light into heat and poor photothermal stability make standalone PTT ineffective [122]. Zou added the anti-angiogenic drug sorafenib to T8IC, an organic semiconductor, using nanoprecipitation to form TS nanoparticles, which generated ROS with high photothermal conversion efficiency [118]. Li designed CuS-Ab NPs loaded with cetuximab to target EGFR to inhibit angiogenesis and tumor growth and to promote the accumulation of CuS NPs in tumors; these NPs reduce the laser energy required for PTT therapy and reduce damage to normal tissue [110]. Cyclic peptide c (RGDfk) can be administered in combination with integrin αvβ3 to inhibit angiogenesis, and Liu constructed cRGD-modified glycolipid-like micelles (cRGD-CSOSA) to overcome the insufficient instability of ICG as a photosensitizer for phototherapy. The binding of cRGD-CSOSA/ICG nanoparticles promotes the production of ROS and improves the efficacy of phototherapy in GBM [112]. Hong bound fibrinogen to AuNPs to generate fAuNPs and used DMXAA to trigger the coagulation cascade in tumor blood vessels to induce the aggregation of fibrinogen. The fAuNPs could thus assemble into insoluble clots in the tumor blood vessels, and given that fAuNPs exhibit absorption peaks in the NIR spectrum, the strategy enhances the photothermal ablation induced by PTT. This combination therapy also reduces the side effects caused by the long-term administration of DMXAA, effectively destroying tumor blood vessels (Figure 4B) [115]. Liang co-delivered CA4 and Prussian blue (PB) in hydrogel for combination anticancer therapy and PTT to induce vascular rupture. PB is activated using near-infrared radiation to strongly attack most cancer cells, and CA4 limits the energy supply; this strategy overcomes inadequate tumor growth suppression due to limited laser penetration depth and provides proof of concept for the “attack + guard” strategy (Figure 4C) [109]. Zhang designed an MCNCD nanoparticle carrying a nonsteroidal anti-inflammatory drug (celecoxib) to inhibit cyclooxygenase-2 (COX-2) from disrupting the PG2I/TXA2 balance, ultimately inducing intravascular thrombosis and reducing the risk of tumor metastasis after PTT treatment. Furthermore, MCNs have high photothermal conversion efficiency, which enhances the PTT effect [111]. Gao’s team developed a nanocarrier that uses near-infrared laser activation. After the near-infrared laser irradiation, the local temperature increase of the nanoparticles in the targeted tumor blood vessels causes the instant rupture of tumor vascular ECs, resulting in the destruction of neovascularization. Photothermal therapy and antivascular therapy are fused to induce the avascular necrosis of tumors in this study [117].

Vascular therapy combined with phototherapy can elicit anti-angiogenic effects to reverse the increase in VEGF seen after phototherapy, target blood vessels, and alleviate the hypoxic microenvironment of tumors to overcome the insufficient ROS production of PDT therapy. Some vascular targeting strategies can also increase the near-infrared absorption of tumors, enabling PTT to kill tumor cells with less laser energy.

## 5. Radiation Therapy

The role and status of radiation therapy in tumor treatment are becoming increasingly prominent, and radiation therapy has become one of the main therapeutic strategies for treating malignant tumors. Tumor hypoxia and high IFP in the microenvironment prevent drugs from easily reaching the tumor, contributing to radiological resistance [123,124]. However, increasing the radiation dose and using radiosensitizers to enhance the effect of radiotherapy can increase toxicity in vivo and damage healthy tissues [125]. Several studies have investigated the potential of combining antivascular therapy and radiation therapy [126,127,128,129]. Yoon et al. conducted a clinical trial in which sorafenib was combined with radiation therapy to improve the overall survival of patients with liver cancer [130]. Zheng designed a heat-sensitive hydrogel (SOR-LUF-SeNPs) that can achieve the local and sustained release of sorafenib within tumors, and the combination of this hydrogel with chemoradiotherapy increased the apoptosis of HepG2 cells in the long-term treatment of HCC [131]. Wang and his team designed Au@SA-QBA, which produces 8HQ in response to H2O2; reduces the expression of VEGF, bFGF, and Ang-2; increases pericyclic-cell coverage and blood flow; normalizes tumor blood vessels; and significantly inhibits tumor growth in combination with radiotherapy [125]. Wang designed a hydrogel loaded with endothelial suppression (ES) that inhibits neovascularization by modulating receptors of angiogenesis factors on the cell membrane, alleviating tumor hypoxia, increasing perfusion to improve drug delivery efficiency, and increasing radiotherapy sensitivity. The systemic toxicity induced by ES can be overcome by administering the drug via injection, which showed excellent antitumor effects in mouse models of Lewis lung carcinoma (LLC) [132]. Some nanoparticles also have their own antivascular effects; for example, Zhang synthesized an H1/pHGFK1 nanoparticle as an angiogenesis inhibitor for GBM therapy; HGFK1 inhibits angiogenesis by regulating the EGFR and bFGF signaling pathways, increasing the resistance of glioblastoma cells to radiotherapy [133]. Tian synthesized a CaBP-PEG nanoparticle that depletes TAMs while inhibiting angiogenesis, correcting the abnormal tumor microenvironment to enhance the effect of cancer radioisotope therapy [134]. Minafra developed a solid nanoparticle (Cur-SLN) containing curcumin that inhibits VEGF and IL-8 and improves the efficacy of radiotherapy [135]. Gold nanoparticles have been shown to inhibit angiogenesis by influencing the expression of growth factors and are effective radiosensitizers [136,137,138]. Yang designed a new gold nanoparticle sensitizer that inhibits HIF-1α-mediated angiogenesis and maximizes the tumor attenuation effects of radiation therapy. In addition, gold nanoparticles enhance the vascular damage caused by VDAs, reducing the oxygen supplied through blood vessels, which results in increased hypoxia and enhances the effect of radiation therapy [139]. Wu designed iron oxide nanoparticles coupled to azademethylcolchicine (CLIO-ICT), a powerful VDA that binds to tubulin and causes a release of serotonin from platelets, disrupting the tumor vascular system. VDA therapy combined with radiation therapy increases radiation sensitivity, and VDAs can kill cancer cells in hypoxic areas with low radiosensitivity to prevent tumor recurrence after radiation therapy [140]. Vascular therapy combined with radiation therapy can improve radiotherapy drug delivery efficiency and radiosensitivity by increasing blood flow perfusion and reducing IFP. In addition, VDAs combined with radiotherapy overcome the insufficient lethality of radiotherapy in tumor sites with low radiation sensitivity.

## 6. Interventional Therapy

Transcatheter arterial embolization (TAE) is a technique that employs the transcatheter vascular injection of embolic agents to occlude a vessel [141]. TAE, in combination with chemotherapeutic drugs (transcatheter arterial chemoembolization (TACE)), is the gold standard for the treatment of unresectable hepatocellular carcinoma (HCC) [11]. It is challenging to achieve total artery embolism with TAE, as one of the most widely used treatments for solid tumors, such as liver cancer; thus, postoperative recurrence and metastasis are common. Some studies have demonstrated that antivascular therapy combined with TAE can achieve the effective long-term embolization of tumor blood vessels, resulting in the ischemic infarction of tumors [142,143]. Shi’s team prepared a TF-nanogel (made from PIB-encapsulated tTF-pHLIPs) that diffuses into the peripheral hepatocellular carcinoma (HCC) vasculature via a temperature-sensitive sol–gel phase transition and thus achieves the embolization of blood vessels. Furthermore, tTF-pHLIPs trigger the coagulation cascade to induce thrombosis formation, thus blocking additional arteries. TAE administered via PIB nanogels and the tTF-pHLIP-mediated vascular blockade strategy have achieved long-term vascular occlusion in rabbit models carrying VX2 tumors, effectively inhibiting the tumor recurrence and metastasis seen with TAE alone [144]. Due to the persistent hypoxic environment of tumors and the high expression of VEGF after TAE treatment, apatinib can inhibit the growth of residual tumors after embolization, because it inhibits VEGFR-2, and can thus achieve the embolization of blood vessels [145]. Zhou used PIB nanogel-loaded apatinib combined with TAE to inhibit the growth of rabbit VX2 liver tumors, overcoming the VEGF overexpression caused by TAE treatment and the issues related to hypoxia after surgery. The study proved that TAE combined with apatinib could be used to continuously embolize blood vessels and improve the long-term efficacy of TAE [146,147].

In radiofrequency ablation (RFA), electrode needles are inserted directly into the tumor or target tissue under the guidance of imaging equipment, generating local heat energy to ultimately induce coagulation in the tumor [148]. However, insufficient RFA treatment promotes tumor angiogenesis and accelerates disease progression [149]. Due to the high vascular density of tumors, substantial heat loss is common, which reduces the efficacy of radiofrequency ablation. A series of studies have proven that RFA, combined with antivascular therapy, is superior to RFA alone [150,151]. Li and her team synthesized a kind of RF-responsive divalent gold nanoclusters, which they administered in combination with TAE and radiofrequency ablation (DV GC@PNA RFA). DV GC@PNA RFA effectively reduced the VEGF overexpression caused by hypoxia after TAE and improved tumor cell sensitivity to heat [152]. Yuan designed a nanocube (Fe_2_O_3_-PDA-Dox) that they combined with CA4P to treat HCC. CA4P increased the permeability of tumor blood vessels and enhanced the effects of TACE combined with photothermal ablation (pTACE) [153].

Vascular therapy combined with interventional therapy, such as TAE, can achieve the long-term occlusion of tumor blood vessels and prevent tumor recurrence and metastasis. Anti-angiogenic drugs can also inhibit VEGF overexpression after TAE treatment and improve sensitivity to the heat generated by RFA.

## 7. Conclusions and Prospects

The growth of solid tumors is highly dependent on tumor neovascularization. The complex vascular network ultimately creates therapeutic resistance, which decreases the effects of single-treatment modalities; challenges include the immunosuppressive state and high IFP of the tumor, which prevent deep tumor penetration by the drugs. In addition, the overexpression of VEGF in the tumor greatly increases tumor resistance. Due to the lack of surrounding cells, the oxygen content of the tumor microenvironment is low, decreasing the effectiveness of aerobic therapy. The appropriate use of nanotechnology to achieve the combination of antivascular therapy and conventional treatment can effectively solve the above problems and improve the efficiency of tumor treatment. In addition, different therapeutic approaches can be combined using nanoplatforms. The effectiveness of tumor treatment has been shown to be maximized in some studies by combining chemoradiotherapy and immunotherapy. This opens up novel therapeutic avenues for cancer patients [154,155]. Gemcitabine (GEM) and 1-methyltryptophan (1MT) amphiphilic biprodrug (GEM-1MT) were self-assembled into nanoparticles by Luan’s team in an aqueous solution to kill tumor cells and reduce immunosuppression in the tumor microenvironment [156]. Using a platinum@polymer-catechol nanobraker, Dai’s team was able to mediate radioimmunotherapy and reduce melanoma’s tumorigenesis, angiogenesis, and radioresistance. 

There are also new treatments being developed for cancer. For example, gene therapy refers to the method of treating diseases by using vectors to transduce exogenous therapeutic genes into cells and then altering the original gene expression of cells with the transcription and translation of exogenous genes [157]. In addition, tumors can be controlled or treated with hormonal drugs [158]. Emerging treatments such as these can bring a new entry point for combination therapy. Nanoplatforms can also provide new ideas for tumor diagnosis. For example, Yang’s team reports a structural and molecular fusion magnetic resonance imaging (MRI) nanoprobe for the differential diagnosis of benign and malignant tumors [159].

If you are a doctor or part of the healthcare community, this review could speed up the process of finding promising cancer treatments. We believe that chemotherapy and immunotherapy are the most widely used pharmacotherapies for tumors in clinical practice and that chemotherapy may also be the first option when cancer spreads and metastasizes. Chemotherapy with antivascular therapy has been slowly introduced to patients. Their synergistic action improves the efficiency of tumor treatment. Additional efficacy can be brought to various targeting regimens with the combination of immunotherapy and antivascular therapy, which is more likely to be clinically available. This is not just a drug stack; the effects of the drugs in this combination therapy complement one another. However, the effectiveness of a treatment plan can vary based on factors such as patient response, drug interactions, and side effects. For this reason, it is important to consider which antivascular drugs may work better in combination therapy. For example, both sorafenib and bevacizumab can be used as antivascular agents for combination immunotherapy, but bevacizumab has been shown to be superior to sorafenib in prolonging progression-free survival in a phase III clinical trial [160,161]. The combination of nano- and antivascular drugs has entered clinical trials. CRLX101 is a nanoparticle–drug conjugate. In a sequential phase II clinical trial, the team found that CRLX101 in combination with bevacizumab improved the objective response rate in recurrent ovarian cancer [162]. In a phase I-IIa clinical trial, CRLX101 in combination with bevacizumab was found to improve progression-free survival in metastatic renal cell carcinoma [163]. In a phase III clinical trial, carboplatin–pegylated liposomal doxorubicin–bevacizumab was found to improve overall survival in patients with recurrent ovarian cancer [164]. However, most of the research related to nanodrug combination therapy strategies is still in the experimental stage and always meets failure when they are put into clinical trials. In terms of biosafety in human bodies, it is particularly important to monitor the complex tumor microenvironment in real time to assess a variety of characteristics related to treatment resistance. In addition, factors such as big-scale manufacturing as well as batch-to-batch consistency are essentially important for the successful translation of the antivascular nanomedicine from bench to bedside. The research and clinical translation of nanomedicines is both a challenge and an opportunity. In recent years, new intelligent antitumor vascular nanodrugs have made significant scientific progress and will likely play an increasingly important role in tumor treatment in the future. Using multiple methods of synergistic therapy is an indispensable treatment strategy for middle and advanced tumors, and the combination of nanomedicine can improve the efficiency of drug delivery, reduce drug side effects, and improve the efficiency of tumor treatment. With the continuous advancement of technology and the deepening of research, we believe that in the near future, more combinations of nanodrugs and antivascular drugs will enter the clinic setting and that they will achieve more excellent therapeutic effects, achieve the long-term and high-quality survival of tumor patients, and bring more hope and confidence to tumor patients.

## Figures and Tables

**Table 1:** Combined therapeutic paradigms of antivascular therapy and immunotherapy.

Antivascular Therapy	Immunotherapy	Disease	Outcomes	Ref.
Axitinib (VEGFR-TKI)	IMT (IDO inhibitor)	Melanoma	Increased proportion of tumor- infiltrating T lymphocytes (CTLs and Th cells); inhibition of Tregs and TAMs	[37]
Bevacizumab (targets VEGF-A/VEGFR-2)	aPD-1 mAb	Colon adenocarcinoma	Increased infiltration by CD8^+^ T cells; upregulated IFN-γ expression; increased amount of aPD-1 mAb delivered to the tumor	[38]
CA4P (ECs)	aPD-1 mAb	Breast cancer	Increases the efficacy of aPD-1 mAb; increase infiltration by CD4^+^ and CD8^+^ T cells	[39]
Tetrathiomolybdate (Inhibits NF-KB signaling.)		Breast cancer	Enhances immune activation	[40]
Endostar (recombinant human endostatin)		NSCLC	Increased IFN-γ and IL-17 expression; decreased TGF-β1 expression	[41]
FSEC (anti-angiogenic peptide)	DPPA (immune checkpoint block peptides)	Breast cancer	Increased infiltration by CD8^+^ T cells	[42]
Gold nanoparticles (inhibit endothelial Smad2/3 signaling)		Gastric carcinoma and breast adenocarcinoma	Increased infiltration by CD3^+^ and CD8^+^ T cells	[43]
pshVEGF-A(VEGF-A)	PshPD-L1	Melanoma	Remission of ICB-induced adaptive resistance	[44]
Sorafenib (multi-target kinase inhibitors)	PD-L1 antibody	HCC	Increases the efficacy of anti-PD-L1 antibodies	[45]

**Table 2:** Combined therapeutic paradigms of antivascular therapy and chemotherapy.

Antivascular Therapy	Chemotherapy	Disease	Outcomes	Ref.
CA-4 (targets ECs)	Dox	Melanoma/breast cancer	Assists chemotherapeutic drugs in eradicating the tumor cells	[68]
CA-4 (targets ECs)	MMP9-DOX	Breast cancer	Induces hypoxia to amplify MMP9 signaling in tumors	[69]
CA-4 (targets ECs)	CDDP	Breast cancer	Increases the retention time to improve the accumulation of drugs within the tumor	[70]
cRGD-folate-heparin nanoparticles (targets endothelium-dependent vessels/antivascular mimicry)	CDDP	Ovarian cancer	Promotes CDDP to effectively inhibit the development and metastasis of cancer	[71]
Curcumin (VEGF) combretastatin A-4 phosphate (VEGFR2)		HCC	Inhibits tumor metastasis	[72]
DA (targets ANG1/VEGF/KL2)	DOX	Breast cancer	Increases blood flow perfusion and reduces IFP	[28]
DMXAA (targets ECs)	DOX	Cervical cancer	Enhances tumor suppression	[26]
Erlotinib (EGFR TKI)	Topotecan	Breast cancer	Prolongs TVN and increases drug delivery efficiency	[73]
Fruquintinib (targets VEGFR-1, -2, and -3)	DOX	Breast cancer	Achieves deep delivery of drugs into tumor tissue	[29]
LMWH (targets bFGF/VEGF)	GA	Breast cancer	Increases blood flow perfusion and reduces IFP	[74]
LMWH (targets bFGF/VEGF)	Paclitaxel/Gemcitabine	HCC	Induces simultaneous drug delivery and normalization of tumor vessels	[75]
Silybin (targets the NF-κB signaling pathway)	Paclitaxel	A549 lung cancer	Chemosensitization	[76]
Thrombin (tumor vessel)	DOX	HCC	Blocks the blood supply to tumors and inhibits cancer cell proliferation	[77]

**Table 3:** Combined therapeutic paradigms of antivascular therapy and phototherapy.

Antivascular Therapy	Phototherapy	Disease	Outcomes	Ref.
Bevacizumab (targets VEGF-A/VEGFR-2)	PDT	Colorectal cancer	Inhibits tumor growth and recurrence	[106]
Candesartan (Ang II receptor blocker)	PDT	HCC	Reduces the secretion of VEGF and restores a normal oxygen microenvironment	[107]
CA4 (targets ECs)	PDT	Breast cancer	Disrupts the vasculature	[108]
CA4 (targets ECs)	PTT	Breast cancer	Restricts the nutrient supply of tumor cells to achieve the “attack + guard” strategy	[109]
Cetuximab (targets EGFR)	PTT	Breast cancer	Decreases the requirement for laser energy and reduces damage to normal tissue	[110]
Celecoxib (targets cyclooxygenase-2)	PTT	Colorectal cancer	Reduces the risk of tumor metastasis after PTT treatment	[111]
cRGD-CSOSA (targets neovascular ECs)	PDT	Glioblastoma	Promotes the production of ROS	[112]
DMXAA (targets ECs)	PDT	Breast cancer	Overcomes the challenges related to hypoxia of traditional type II PDT and inhibits tumor metastasis	[113]
DMXAA (targets ECs)	PTT/PDT	Cervical cancer	Achieves complete tumor ablation	[114]
DMXAA (targets ECs)	PTT	Colon cancer	DMXAA promotes aggregation of gold nanoparticles with NIR absorption to increase absorption and enhance the photothermal ablation of PTT	[115]
HBA (targets VEGF)	PTT/PDT	Colorectal cancer	Reduces the secretion of VEGF	[116]
Infrared laser irradiation (ECs)	PTT	Cervical cancer	Induces avascular necrosis of tumors	[117]
Sorafenib (VEGFR/PDGFR TKI)	PTT/PDT	OSCC	Increases photothermal conversion efficiency and ROS production	[118]
TNP-470 (VEGF)	PDT	Prostate carcinoma	Effectively reduces tumor growth and metastasis	[97]

## Data Availability

Not applicable.
